# Vitreous Inflammation Associated with Intravitreal Anti-VEGF Pharmacotherapy

**DOI:** 10.1155/2013/943409

**Published:** 2013-11-06

**Authors:** Shivi Agrawal, Malav Joshi, John B. Christoforidis

**Affiliations:** ^1^Department of Ophthalmology, University of Arizona Medical Center, Tucson, AZ 85711, USA; ^2^Retina Division, Department of Ophthalmology, University of Arizona Medical Center, 655 N. Alvernon Way, Suite 108, Tucson, AZ 85711, USA

## Abstract

Vascular endothelial growth factor (VEGF) is a potent promoter of angiogenesis involved in a wide variety of physiologic processes. Intravitreal injections targeting VEGF have transformed the treatment of neovascular retinal diseases. Currently, there are four anti-VEGF agents in use: bevacizumab, ranibizumab, pegaptanib, and aflibercept. The success and frequency of anti-VEGF therapy have made the ocular safety profile of these agents of vital importance. This paper focuses on sterile endophthalmitis. In this paper, we compare the incidences of posttreatment sterile endophthalmitis among the four agents, review the mechanism of actions, and discuss the most prevalent hypotheses leading to sterile endophthalmitis.

## 1. Introduction

Vascular endothelial growth factor-A (VEGF) is the master regulator of angiogenesis [1]. Pharmacotherapy utilizing intravitreal injections of antivascular endothelial growth factor (anti-VEGF) agents has revolutionized the treatment of neovascular retinal disorders by inhibiting angiogenesis. Bevacizumab was the first intravitreal agent utilized for the treatment of macular edema secondary to a branch retinal vein occlusion and age-related macular degeneration [2, 3]. Today, multiple anti-VEGF agents have been developed including bevacizumab, pegaptanib, ranibizumab, and aflibercept. These agents have shown promising results in the treatment of various retinal diseases including age-related macular degeneration, diabetic retinopathy [4], neovascular glaucoma [5], retinopathy of prematurity [6], and intraocular tumors [7]. Today, the use of intravitreal anti-VEGF agents is the most common intravitreal procedure performed by ophthalmologists. The recognition of adverse effects from the use of these medications and appropriate treatment has become increasingly important. In this paper, we will compare the inflammatory effects of the different anti-VEGF agents, differentiate their clinical features, and review the possible mechanisms involved in the development of posttreatment sterile inflammation. 

## 2. Definition of Sterile Endophthalmitis

Sterile endophthalmitis (also known as “pseudoendophthalmitis”) is described as any acute intraocular inflammation without infection that resolves without antibiotic treatment, unlike true endophthalmitis. A review of the literature published on PubMed between 1945 and June 2013 was conducted using combination keywords such as sterile endophthalmitis, anti-VEGF, pegaptanib, bevacizumab, ranibizumab, aflibercept, and ocular inflammation. Only the articles written in English were included. Also, in order to avoid confusion, only the studies reporting noninfectious endophthalmitis were included. 

## 3. Sterile Endophthalmitis versus Infectious Endophthalmitis

Infectious endophthalmitis is the most feared complication after intravitreal injections. It is important to differentiate infectious endophthalmitis from sterile endophthalmitis, as the management and prognosis of these two entities vary vastly. While infective endophthalmitis cases are heavily treated by intravitreal antibiotics, the treatment of sterile endophthalmitis has shown prompt improvement with topical steroid therapy [8]. The clinical features can help when attempting to differentiate the two (Table 1).

In the literature, the incidence of sterile endophthalmitis after intravitreal anti-VEGF therapy ranges between 0.033% and 2.9% [9–14]. Meta-analyses reports have shown variability in the incidence of sterile endophthalmitis between the different anti-VEGF agents (Table 2). It typically presents 24 hours to 7 days after injection [10, 15], with or without pain. Pain may be an indication of the severity of the inflammation in the anterior chamber and vitreous cavity. The most common presenting symptoms are blurred vision and floaters [11]. The time between symptom presentations after injection ranges from 1 day to 1 week [10, 11, 14–18]. Visual acuity at presentation is substantially reduced compared with preinjection acuity and typically returns to preinjection acuity after resolution of the inflammation [10, 11]. The average time to resolution of inflammation ranges from 2 to 12 weeks [11, 15, 17] and recovery of visual acuity occurs between 7 and 9 weeks [11]. Moreover, the time from injection to presentation with inflammation does not seem to affect the extent of visual recovery; it only affects the length of time to recovery [10]. In addition, history of prior intravitreal anti-VEGF injections does not increase the risk or severity of ocular inflammation in subsequent injections [11, 19].

The clinical course of sterile endophthalmitis varies based on the management of the clinical practitioner. Management includes the use of topical medications, intravitreal antibiotics, and pars plana vitrectomy with or without intravitreal antibiotics. The time to resolution based on this can vary from two to 42 days (Table 1). The median duration of inflammation was six days in patients undergoing vitrectomy, seven days in patients receiving triple intravitreal injections, and four days in patients receiving topical corticosteroids [24]. While these results may imply that treatment with topical corticosteroids is the most effective, this is not accurate. Chong et al. [11] reported only 0.27%, 14 of 16166 cases, of sterile endophthalmitis resolving with topical antibiotics alone. It is difficult to generalize the treatment with the time to resolution because typically the most severe cases were chosen for pars plana vitrectomy with intravitreal antibiotics. Shah et al. [15] reported in a retrospective case series that the clinical difference between these two entities was not significant and therefore a low threshold for vitreous tap with intravitreal antibiotic injection might be warranted. 

## 4. Pharmacokinetics of Anti-VEGF Agents

Before discussing possible mechanisms of inflammation after intravitreal injection, it is important to examine the pharmacokinetic properties of these agents, especially in regard to the duration of activity within the vitreous. The intravitreal half-lives of bevacizumab using ELISA methods range from 4.32 to 9.82 days [41–43]. Similarly, the intravitreal half-life of ranibizumab was approximately 7.15 days [44]. In a rhesus monkey study, the vitreous half-life of pegaptanib was found to be approximately 3.9 days [45]. There are currently no reports on the pharmacokinetic properties of aflibercept using ELISA. Utilizing PET/CT to detect I-124 labeled anti-VEGF agents, Christoforidis et al. reported the intravitreal half-lives of ranibizumab, bevacizumab, and aflibercept [46, 47] to be 2.82, 4.22, and 4.58 days, respectively. Their findings corroborate the previously described presence of a two-compartment pharmacokinetic decay model with an initial rapid phase followed by a slower phase described by Zou et al. [48]. 

## 5. Antivascular Endothelial Growth Factor Drugs

Targeting anti-VEGF in the treatment of ocular neovascular diseases first requires an understanding of the human VEGF-A gene. The human VEGF-A gene is composed of eight exons with six principle amino acid isoforms (121, 145, 165, 183, 189, and 206) [1]. VEGF121 is freely diffusible, while VEGF189 and VEGF206 are primarily bound and sequestered in the extracellular matrix. This is due to the heparin-binding domain found in the larger isoforms of VEGF such as VEGF189 and VEGF206. VEGF165 has properties of both the diffusible and bound form of VEGF [1]. There are four anti-VEGF agents currently utilized in the treatment of ocular diseases, which differ in their isoform-binding specificities. 

Pegaptanib (Macugen; Eyetech/OIS Pharmaceuticals, Melville, New York, USA) was the first anti-VEGF therapy approved for the treatment of wet AMD. It is an aptamer that selectively binds to and neutralizes VEGF-A165 while sparing smaller isoforms that lack the heparin-binding domain such as VEGF121 and VEGF110 [49]. The large-scale, randomized controlled VISION trials reported twelve cases of 1190 cases of endophthalmitis (1%) although nine of the twelve were likely associated with violations of the injection preparation protocol, such as failure to use an eyelid speculum. In year 2, there were four cases in 1024 patients of endophthalmitis (0.4%) [39]. There were no reported cases of sterile endophthalmitis [39, 40]. 

Ranibizumab (Lucentis; Genentech, South San Francisco, California) was designed as a potent inhibitor of all VEGF isoforms with an affinity-matured antigen-binding fragment (Fab) derived from bevacizumab. It was developed as a Fab fragment because it was thought that its smaller-size would increase its diffusion capacity as an anti-VEGF-A agent compared to its parent bevacizumab. Ranibizumab, compared to its parent bevacizumab, has a higher affinity for VEGF with a greater potency. As an antibody-binding fragment, it lacks the domain that activates compliment-mediated cytotoxicity and Fc receptors on immune cells [50]. The primary ocular adverse event associated with ranibizumab is ocular inflammation. Many large-scale studies have reported the frequency of ocular inflammation or presumed endophthalmitis to be between 0 and 12.7%. The results of these trials have been outlined below. 

The rate of intraocular inflammation in the MARINA trial using ranibizumab was found to be 2.6% [12]. In the PIER study, no cases of serious uveitis or endophthalmitis were noted with the use of ranibizumab [28]. In the IVAN trial, only one case of 610 (0.1%) developed uveitis [34]. The HARBOR study group reported a 0.7% rate of endophthalmitis and a 0.4% rate of inflammation with intravitreal ranibizumab [30]. The SUSTAIN study utilized ranibizumab and reported no episodes of sterile endophthalmitis in the 249 patients studied [29]. The first year results of the FOCUS study revealed that the more frequently associated serious ocular adverse events were intraocular inflammation (11.4%) and endophthalmitis (1.9%; 4.8% including presumed cases) [31]. In the two-year FOCUS study, endophthalmitis and serious ocular inflammation occurred in 2.9% and 12.4%, respectively, in the ranibizumab + PDT patient groups [32]. It should be noted that this study used a lyophilized formulation of ranibizumab that was discontinued afterwards. Among the 280 patients treated in the ANCHOR trial, presumed endophthalmitis occurred in 2 patients (0.7%) and serious uveitis occurred in 1 patient (0.35%) at year 1 [13]. Ladas et al. reported a 1.9% frequency of ocular inflammation after intravitreal ranibizumab and bevacizumab injection with no reported statistical difference between the two medications [35]. Overall, the rates of presumed endophthalmitis or severe inflammation were similar between the two drugs.

Bevacizumab (Avastin; Roche, Basel, Switzerland) was initially a drug approved by the Food and Drug Administration for the treatment of glioblastoma, metastatic colon cancer, advanced nonsquamous non-small cell lung cancer, and metastatic kidney disease. It is a full-length murine-derived humanized, monoclonal, nonselective antibody against VEGF-A. It is a significantly larger molecule with potentially less effective retinal penetration and binding affinity to VEGF. In comparison to ranibizumab (Lucentis), which is an antibody fragment, bevacizumab has an Fc fragment which may make it more immunogenic or proinflammatory. Larger molecules with Fc constant fragments and antibody-binding Fab fragments are more immunogenic than those with the antibody-binding fragment alone. 

A retrospective single center study conducted by Johnson et al. reported the incidence of intraocular inflammation after bevacizumab injection to be 1.3% after 693 injections [26]. Similarly, Georgopoulos et al. reported a 0.3% incidence of intraocular inflammation after 2500 injections of bevacizumab [25] and Shima et al. reported a 0.2% incidence of ocular inflammation after 1300 injections [16]. In a smaller study that used the same lot of bevacizumab, 5 of the 35 (14.3%) of the patients developed severe intraocular inflammation [22]. In this study however, 80% of the patients had received bevacizumab injections previously without an intraocular inflammatory episode. A similar incidence of lot specific intraocular inflammation has been reported in China where 80 patients of 116 (69%) developed postinjection intraocular inflammation [24]. This study implicated endotoxin as the cause of the inflammation. 

Aflibercept (Eyelea, Regeneron, Inc., Tarrytown, NY) utilizes the fusion of multiple endogenous receptor components creating what is called a VEGF Trap. It binds with higher affinity to multiple isoforms of VEGF-A as well as VEGFR1 ligands, VEGF-B, and placental growth factor (PIGF). Consistent with this higher affinity, VEGF Trap demonstrates a higher ability in blocking VEGF-mediated mobilization and migration of human endothelial cells [51].

It was approved by the FDA in November 2011, and within the first three months after its approval, a cluster of small cases were reported with injection-related ocular inflammation [37]. This report indicated that aflibercept was associated with sterile inflammation in 0.05% of cases and associated with pain far more than the other anti-VEGF agents (60%). Prior to approval of aflibercept, the clinical characteristic of pain could often be used to distinguish between sterile inflammation and endophthalmitis. This report has led clinicians to be more cautious and more apt to treat with intravitreal antibiotics sooner. Subgroup analysis in this study did not detect any variables significantly affecting visual outcome or number of days to resolution. Moreover, Ho et al. looked at the short-term outcomes of aflibercept in 245 patients for five months and reported no cases of endophthalmitis [38]. More recently, The American Society of Retina Specialists Therapeutic Surveillance Committee (ASRS TSC) reported at the annual ASRS meeting in August 2013 that there were at least 41 cases of sterile endophthalmitis among more than 800,000 aflibercept injections given in the United States between December 2011 and June 2013. While some of these reported cases responded to topical corticosteroid treatment and observation alone, others were associated with more severe inflammation that resembled infectious endophthalmitis. The ASRS TSC concluded that there was no clear pattern detected to predict these events.

## 6. Hypotheses

There are several hypotheses pertaining to the etiology of sterile inflammation secondary to intravitreal anti-VEGF injections. The manufacturer's guidelines for anti-VEGF agent preparation state that the medication should be refrigerated at 2 to 8 degrees C (36 to 46 degrees F), protected from the light, stored in the original carton until used, and used within 8 hours of being opened [10]. Any variance from this protocol could result in degradation of the agent with increased immunogenicity [52, 53]. 

The eye may mount an immune response to the antibody molecule after prior exposure to the drug. One report found an 83% incidence of sterile inflammation after intravitreal injection of ranibizumab. One of the 29 patients (0.03%) had to be permanently withdrawn from the study secondary to the severe inflammation. In this study, the inflammation was low-grade and self-limited and did not increase with repeated injections or increasing doses [18]. While these results do not support this immune mediated hypothesis, the immunogenicity varies between different anti-VEGF agents. 

Bacterial endotoxin contamination has been reported in the pharmaceutical production phase of antibody preparation [24, 54]. In a study by Wang et al., a total of 116 patients were injected from 3 vials of counterfeit bevacizumab with 80 patients subsequently developing intraocular inflammation. The presence of endotoxin in vitreous specimens was confirmed by laboratory testing. They concluded that endotoxin testing should be considered as part of the laboratory investigation in patients who develop noninfectious inflammation. These studies demonstrated that endotoxin contamination of individual aliquots is possible during preparation. While this occurrence could explain clusters of sterile endophthalmitis cases in patients treated with injections from the same batch, it is unlikely to explain the cause of sporadic cases. 

While systemic use of anti-VEGF agents has not been described to entice an inflammatory response, in the closed system of the eye, it may mount a significant response to the anti-VEGF. Multiple case series have described a high percentage (35–78%) of sterile ocular inflammation after intravitreal injections from a single lot of anti-VEGF agents. 

Yamashiro et al. reported 14 consecutive cases of endophthalmitis after intravitreal injection of bevacizumab from the same lot. Of the 19 eyes, 14 showed ocular inflammation after injection. Vitreous samples from these patients revealed the etiology to be noninfectious [19]. Similarly, in a report by Sato et al., 14.3% (five of 35 cases) were noted to develop a severe intraocular inflammation after intravitreal injection of bevacizumab. Vitrectomy was performed in all 5 cases with no growth of any causative organisms or microbes [22]. All five cases were resolved with treatment with steroids. These reports support the possibility of trace endotoxin contamination resulting in sterile endophthalmitis. Wang et al. published a retrospective paper where 116 patients were injected with counterfeit bevacizumab. Of these patients, 69% developed a sterile endophthalmitis with endotoxin levels (endotoxin units) detected as high as 36 Eu/mL (standard of bevacizumab <2 Eu/mL) [24]. These patients shared the typical clinical features described above with 78% of the affected patients returning to pre-injection visual acuity. 

In summary, sterile inflammation is an adverse event of intravitreal anti-VEGF injection that should be included in the patient consent in all anti-VEGF agents. Acute intraocular inflammation is most frequently following bevacizumab [36], possibly due to the less stringent purification process of the medication. In most cases, the inflammation resolves and vision returns to baseline. A history of prior inflammation does not increase the risk with subsequent injections.

## Figures and Tables

**Table 1:** Clinical characteristics of noninfectious versus infectious endophthalmitis.

	Noninfectious endophthalmitis	Infectious endophthalmitis
Pain	± [14, 17, 20]	++ [14]
Onset	<1 day [10, 14, 16, 20, 21] to 1 week [11, 15, 17, 22]	2.5 days (range: 1–6 days) [14, 15, 23]
Signs	Blurred vision [11], anterior segment inflammation greater than posterior inflammation [10, 17, 18, 21, 22]	Decreased vision, severe anterior segment reaction (fibrin and hypopyon), and vitritis [14]
Time to resolution	2–12 weeks [11, 15, 17, 18, 20, 24]	Extremely variable
Prognosis	Preinjection visual acuity [10, 11, 14, 16–18, 21, 22]	Severely depressed [22]

**Table 2:** Sterile inflammatory rates between anti-VEGF agents.

Study	Anti-VEGF agent	Number of patients	Number of injections	Percentage (%) of inflammation
Chong et al. (2010) [11]	Bevacizumab	—	16116	0.40%
Georgopoulos et al. (2009) [25]	Bevacizumab	—	2500	0.03%
Shima et al. (2008) [16]	Bevacizumab	707	1300	0.28%
Wickremasinghe et al. (2008) [10]	Bevacizumab	—	1278	1.49%
Johnson et al. (2010) [26]	Bevacizumab	173	693	1.30%
Sato et al. (2010) [22]	Bevacizumab	35	35	14.3%
Yamashiro et al. (2010) [19]	Bevacizumab	15	20	73%
Wang et al. (2013) [24]	Bevacizumab	116	116	69%
Wu et al. (2008) [27]	Bevacizumab	1173	4303	0.09%
Chong et al. (2010) [11]	Ranibizumab	—	3839	0.03%
Regillo et al. (2008) [28]	Ranibizumab	184	—	0%
Holz et al. (2011) [29]	Ranibizumab	514	—	0%
Busbee et al. (2013) [30]	Ranibizumab	1098	—	0.4%
Rosenfeld et al. (2006) [12]	Ranibizumab	716	—	2.6%
Brown et al. (2006) [13]	Ranibizumab	280	—	0.35%
Heier et al. (2006) [31]	Ranibizumab	105	—	11.4%
Antoszyk et al. (2008) [32]	Ranibizumab	105	—	9.5%
Rosenfeld et al. (2006) [18]	Ranibizumab	29	—	86%
Chun et al. (2006) [33]	Ranibizumab	10	30	50%
Chakravarthy et al. (2012) [34]	Bevacizumab and ranibizumab	610	—	0.16%
Ladas et al. (2009) [35]	Bevacizumab and ranibizumab	450	2000	1.90%
Sharma et al. (2012) [36]	Bevacizumab and ranibizumab	524	1584	1.90%
Hahn et al. (2013) [37]	Aflibercept	—	30000	0.05%
Ho et al. (2013) [38]	Aflibercept	85	—	0%
D'Amico et al. (2006) [39]	Pegaptanib	1190	—	0%
Singerman et al. (2008) [40]	Pegaptanib	161	1254	9%
